# The Faradic Coil in Gynecological Practice1Read to the Buffalo Medical and Surgical Association, January 5, 1892

**Published:** 1892-03

**Authors:** Herman E. Hayd

**Affiliations:** M. D., M. R. C. S., Eng., Buffalo, N.Y.; 78 Niagara Street


### THE FARADIC COIL IN GYNECOLOGICAL PRACTICE.[1]

By HERMAN E. HAYD, M. D., M. R. C. S., Eng., Buffalo, N. Y.

In the August number of the *Post-Graduate* there appeared a paper by Dr. Bache Emmet, as well as the discussion by many of the most prominent gynecologists in New York City, which serves to show the interest the profession is taking in electro-therapeutics. It is but a few years ago, and certainly within the recollection of most of you, that these same men looked with surprise and even contempt upon those reputable and scientific men who were wasting so much time and energy over that so-called dangerous plaything. And yet, as Emmet says, there is hardly a gynecologist in New York City, or in any city in the United States, who is not using this valuable agent in his every-day routine work with benefit and satisfaction in properly selected cases.

The possibilities of the galvanic current are no doubt familiar to you, since so much has been written upon it during the past few years; but the literature, as well as the conclusions in reference to the special uses of the faradic, are still unsettled. To Apostoli is due the credit of not only giving the galvanic current its scientific importance, and its wide application in special forms of disease, but he has also, by much labor and experiment, developed the properties and uses of the faradic. He found that the more the wire of the secondary coil was attenuated, the more sedative were the properties of the current generated, consequently he was led to use it in the treatment of acute and subacute inflammation of the uterus and its appendages. Unfortunately there is no guide or standard in the manufacture of faradic batteries, and in order to get actual and uniform results from relatively different sized wires, the construction of the battery must have your special study and consideration. Engelmann's coils are, on the whole, very satisfactory. His number one coil is made of number sixteen wire, and seventy-five yards in length; (2) twenty-two wire, 225 yards in length; (3) number thirty-two wire, 660 yards in length, Brown & Sharpe American scale. You will find in most batteries, and particularly the cheaper ones, coarse wire from fifteen to thirty, and about

1. Read to the Buffalo Medical and Surgical Association, January 5, 1892

sixty feet to 200 yards in length; the principal motive on the part of the manufacturer being to give a loud, noisy, powerful machine. This brings us to a second consideration, namely, the vibrator, which should be easy and simple in adjustment, and capable of making very fine and very rapid vibrations. When the coarse wire is employed, it is with the expectation of giving tone and strong muscle contractility; therefore the vibrator should be slow, so as to make the muscle alternately contract and relax itself; but when the fine wire is used, it is with the expectation of relieving pain, and producing sedation in excitable nerve areas and irritable muscle-contractions. When the interruptions are rapid, the muscles are unable to respond to every vibration, and a constant contraction or a tetanoid condition is produced, which eventually wears out this contractile power. The same constant and intense stimulation of the sensory nerves results in a temporary loss in their power to respond; and, as a consequence, a condition of anesthesia is produced, more or less prolonged, depending upon the duration and frequency of the applications. With the "Goelet" coarse wire, the vibrations should be from fifty to eighty per second, while with the fine wire they should be from 150 to 200 per second.

I have been especially pleased with the faradic coarse coil, or coil of quantity, in recent subinvolution following abortion, when slight hemorrhage continues for days, and even weeks, due to a want of tonic contractility in the uterine muscle-fibre. Ergot and other uterine stimulants are often insufficient to control this condition, and there soon results, from a simple inertia, a possible chronic subinvolution, with fungous endometritis, salpingitis, and all their attending complications. Moreover, after too frequent child-bearing, or in conditions of debility accompanying recent parturition, when the womb lacks its proper contractile power, and, as a result, absorption and involution are impeded, the faradic current, combined with general tonic treatment, will often afford the most satisfactory results. These treatments can be taken by the patient herself, and require simply a clay abdominal pad and a vaginal electrode, and as much electricity, gradually turned on, as the patient can bear. The séance should last about ten minutes, two or three times a week. Cases of this kind I reported in the December 19th number of the *New York Medical Record*, in a paper on the Treatment of Uterine Hemorrhage. You will also find that these cases are often complicated with vaginal subinvolution when the canal remains large and its muscular walls are relaxed and flabby, a condition in which you would expect, and, in fact, will surely find benefit from the application of the coarse faradic coil with slow interruptions. Moreover, all cases of simple pelvic congestion, when the hemorrhoidal vessels, and the pampiniform plexus of vessels in the broad ligament are over-distended, and when there is more or less sagging or dropping down of the whole pelvic contents, with settling down of the bowel itself, and, perhaps, constipation, with a relaxed perineum, are often quickly relieved by this form of electricity.

But the fine coil, or coil of tension, is what I wish particularly to draw your attention to, because it possesses such powerful sedative properties, which can be utilized for the relief of pain, which is the most prominent symptom in connection with so many forms of womb trouble, an irritable or hyperesthetic nervous supply, or from more or less acute inflammatory exudates in the womb itself, or its adjacent tissues. I have reported in the *Archives of Pediatrics, Obstetrics and Gynecology*, September, 1891, some interesting cases, where the results were very gratifying. You will find it especially valuable in those acutely retroflexed wombs where the body is so hypersensitive that even the slightest pressure upon the mass gives excruciating pain, and where the vaginal walls themselves are tender to the touch; in inflamed, tender, and often prolapsed ovaries, accompanied with much pain in the iliac fossa, pain and sickness in the pit of the stomach; also in some neuroses, vaginismus when an operation is not called for, and certain irritable conditions of the nervous mechanism of the bowels, where flatulence, roaring, or croaking noises seriously disturb the comfort and happiness of some women. In one case, that of a young, unmarried woman, in whom I had tried every medicine known to me without effect, and where the condition was so exaggerated that she was denied the pleasure of society, and was becoming a hopeless hypochondriac, I found both ovaries tender and sensitive to the touch, with marked vaginismus, and, after a few treatments, ten in all I think, together with a good tonic course of medicine, which had been given the patient for some weeks previous, a perfect cure was effected.

It is, moreover, a valuable agent to commence the treatment of painful conditions; in fact, to supplement galvanism, vaginal or intrauterine, when indicated.

It has one great advantage in being practically without danger, unless carelessly administered in acute inflammations, since intrauterine manipulation is uncalled for, unless under very special circumstances, when it may be found advisable to use it intra-uterine. Care should always be exercised to commence a painful case with a very fine wire and a long coil ; have a clean, easily adjusted, rapid vibrator ; commence with the weakest possible current, and gradually, without shock or interruption, increase it, and reduce it with the same care, and continue the treatment for fifteen minutes, when you will often find complete anesthesia of a previously painful, highly sensitive mass.

Let me report an interesting case, not previously recorded, since it is a young woman who has been under the care of many of you, and who finally saw nothing before her but laparatomy for the possible relief of her suffering. I also show for your inspection ten different pessaries worn by this young woman, which demonstrates conclusively how uncalled for pessaries are in many cases so frequently treated by them. In fact, I am of the opinion that not one in ten pessaries that are employed nowadays in the average general practitioner's practice can do anything but harm.

S. D., *æt.* twenty-eight ; unmarried ; well nourished ; brunette. Menses first appeared when twelve years of age, with great pain ; regular for some years, and periods lasted three days ; flow never profuse, and came every twenty-eight days. She consulted me on April 4, 1891, complaining of great pain when unwell, in fact, so violent that she could not lie down in bed, but would roll and writhe in agony, and scream from the violence of her suffering for at least twenty-four hours if morphine were not administered ; constant and protracted retching, and often vomiting of blood. Bloating, and violent pains in the breasts. For the last three years periods have come every twenty to twenty-three days, and during the whole crisis she would see but a few drops of blood. During the interval she complained of having dorsal pain, pains in the groins, and excessive backache, with constant desire to stool, as well as frequency and precipitancy in urination ; extremely nervous and hysterical, suffering much from globus and throat constriction ; in fact, a physical wreck. Locomotion painful, and she would never attempt to walk more than a few squares. She stooped to the left side, and the dresses were always first worn out on the left side. No pain on urination, but great distress after the act ; nocturnal desire, and she would be compelled to get up three or four times a night. Some discharge ; bowels costive.

Diagnosis : Acute retroflexion with considerable fixation ; enlargement. Some thickening in both ligaments, especially the left, where the ovary could be felt extremely tender and enlarged. General congestion of cervix ; endocervicitis ; endometritis ; *hysteria.*

The examination caused considerable pain, but not from acute inflammatory exudate, but a general hyperesthetic condition of the parts. The patient was given a treatment with the fine faradic coil, number thirty-two, 2,884 feet long, for fifteen minutes, with the effect of materially lessening the pain immediately upon examination. Six Léclanche cells were used. The vagina was tamponned with a ten per cent. solution of ichthyol in glycerine, and a mixture given of tinc. valer. ammon., M. xv., sp. ether chlor. M. v., every fourth hour. The treatments were continued every other day, with the effect of gradually overcoming all the pain and tenderness upon examination. Then Goelet's clay-ball electrode, positive galvanism fifty ma., for seven minutes was applied three times a week to the retroflexed mass and vaginal vault, and finally the negative pole, with the expectation that it would more speedily dissipate the exudate, and possibly lessen the adhesions. After each treatment the tampons were again applied well behind the os. Up to May 23d it was with difficulty that I persuaded the patient of the improvement in her local condition, since her general symptoms were those of discomfort and suffering ; but on June 15th she reported : Menses came on the twenty-fifth day, the first time in three years at such a long interval, with some pain, but didn't take to bed ; was feeling very well ; her despondency and unnatural forebodings were passing away ; was in good spirits ; walked from North street without pain, and for the past two days didn't know what pain was, while heretofore never knew what it was to be free from it. The treatments were continued twice a week for two months longer, when the patient felt comfortable enough to remain away, which she has done for the past three months ; reporting after each period. Upon the last examination, October 25th, the womb was found to be free from pain upon extreme manipulation, the flexion was much improved, in fact, the body was simply directed posteriorly, and her general condition was satisfactory. Some adhesions still existed, but no tenderness.

Now, what constitutes a cure ? Must the womb be anatomically in a certain angle, or in a definite line, which shall run from the umbilicus to the base of the spine ? No ; but the acute and subacute congestion, as well as inflammation and the inflammatory adhesions, which caused so much pain and tenderness, and irritative disturbance, must, to a very great extent, disappear; then, nature will tolerate, as she usually does, a slightly misplaced womb, when this malposition is not accompanied by irritable parametritic deposits. Moreover, by desisting from needless intrauterine medication, the introduction of unnecessary foreign bodies in the shape of electrodes, sounds, stem pessaries and ordinary pessaries, which were uncalled for, at all events in her case, no irritative phenomena resulted from the applications; and, as a result, there was uterine sedation and physiological rest, affording the best possible means for the absorption of the inflammatory products. The periods were associated with but a moderate degree of pain, and were somewhat increased in amount, and the vesical disturbance, as well as the general reflex irritative symptoms of the stomach and other organs, disappeared. Normal and equable discharges of nerve force took place, and instead of a broken-down, shattered, and hysterical nervous system, brought about by constant ovarian irritation, one of healthy and settled function resulted. The patient is in good health and spirits, increased in flesh, and to all intent and purpose a well woman.

78 NIAGARA STREET.

